# Which factors prognosticate spinal instability following lumbar laminectomy?

**DOI:** 10.1007/s00586-012-2250-y

**Published:** 2012-03-17

**Authors:** Arno Bisschop, Barend J. van Royen, Margriet G. Mullender, Cornelis P. L. Paul, Idsart Kingma, Timothy U. Jiya, Albert J. van der Veen, Jaap H. van Dieën

**Affiliations:** 1 Department of Orthopedic Surgery, VU University Medical Center, Research Institute MOVE, De Boelelaan 1117, P.O. Box 7057, 1081 HV Amsterdam, The Netherlands; 2 Faculty of Human Movement Sciences, Research Institute MOVE, VU University Amsterdam, Van der Boechorststraat 7, 1081 BT Amsterdam, The Netherlands; 3 Department of Physics and Medical Technology, VU University Medical Center, De Boelelaan 1118, 1081 HV Amsterdam, The Netherlands

**Keywords:** Degenerative spondylolisthesis, Decompression, Shear biomechanics, Spinal stenosis and diagnostics

## Abstract

**Purpose:**

Reduced strength and shear stiffness (SS) of lumbar motion segments following laminectomy may lead to instability. The purpose of the present study was to assess a broad range of parameters as potential predictors of shear biomechanical properties of the lumbar spine.

**Methods:**

Radiographs and MRI of all lumbar spines were obtained to classify geometry and degeneration of the motion segments. Additionally, dual X-ray absorptiometry (DXA) scans were performed to measure bone mineral content and density (BMC and BMD). Facet sparing lumbar laminectomy was performed either on L2 or L4, in 10 human cadaveric lumbar spines (mean age 72.1 years, range 53–89 years). Spinal motion segments were dissected (L2–L3 and L4–L5) and tested in shear, under simultaneously loading with 1600 N axial compression. Shear stiffness, shear yield force (SYF) and shear force to failure (SFF) were determined and statistical correlations with all parameters were established.

**Results:**

Following laminectomy, SS, SYF, and SFF declined (by respectively 24, 41, and 44%). For segments with laminectomy, SS was significantly correlated with intervertebral disc degeneration and facet joint degeneration (Pfirrmann: *r* = 0.64; Griffith: *r* = 0.70; Lane: *r* = 0.73 and Pathria: *r* = 0.64), SYF was correlated with intervertebral disc geometry (*r* = 0.66 for length; *r* = 0.66 for surface and *r* = 0.68 for volume), BMC (*r* = 0.65) and frontal area (*r* = 0.75), and SFF was correlated with disc length (*r* = 0.73) and BMC (*r* = 0.81). For untreated segments, SS was significantly correlated with facet joint tropism (*r* = 0.71), SYF was correlated with pedicle geometry (*r* = 0.83), and SFF was correlated with BMC (*r* = 0.85), BMD (*r* = 0.75) and frontal area (*r* = 0.75). SS, SYF and SFF could be predicted for segments with laminectomy (*r* ^2^ values respectively: 0.53, 0.81 and 0.77) and without laminectomy (*r* ^2^ value respectively: 0.50, 0.83 and 0.83).

**Conclusions:**

Significant loss of strength and SS are predicted by BMC, BMD, intervertebral disc geometry and degenerative parameters, suggesting that low BMC or BMD, small intervertebral discs and absence of osteophytes could predict the possible development of post-operative instability following lumbar laminectomy.

## Introduction

Lumbar laminectomy is a commonly used treatment for symptomatic degenerative lumbar spinal stenosis [6]. Although the impinged nerves are decompressed and neurological symptoms, such as low back pain, sciatica, claudication, motor, sensory and reflex activity, often improve following lumbar laminectomy, it can lead to symptomatic postoperative lumbar instability or even postoperative failure of the spinal motion segment [14]. A well-known complication of lumbar laminectomy is excessive shear displacement in the intervertebral joint, leading to postoperative spondylolysis or spondylolisthesis [7]. Symptomatic clinical instability justifies reoperation to stabilize and fuse the unstable segment [8]. When residual strength and shear stiffness (SS) of the lumbar spine after laminectomy can be predicted, this may support patient selection for additional spinal stabilization. In other words, based on predicted residual shear properties, the surgeon may decide whether or not to combine laminectomy with (instrumented) fusion techniques.

Previously, we showed in an in vitro experiment that laminectomy resulted in a substantial decrease of SS and shear force to failure (SFF) of lumbar spinal segments [1]. However, the biomechanical behaviour of a spinal motion segment following laminectomy will likely also depend on disc degeneration, facet joint degeneration, Modic changes, Schmorl’s nodes, intervertebral disc and pedicle geometry, and facet joint angles.

To our best knowledge, there is a lack of information in literature, demonstrating correlations between these various anatomical and clinical parameters and the biomechanical behaviour of a spinal motion segment following lumbar laminectomy. In this study, we aim to assess the relationship between various anatomical and clinical parameters, and in vitro strength and SS of a lumbar spinal segments either untreated or following facet sparing laminectomy. A total of ten spines (Th12–L5) were used. Ten segments remained untreated (five times L2–L3 and five times L4–L5) and ten segments were treated with laminectomy (five times L2–L3 and five times L4–L5).

We hypothesized that multiple independent variables, together, determine shear biomechanics of a lumbar spinal segment either intact or treated with laminectomy. Identification of these determinants may enable prediction of shear biomechanics in the future, which may support surgical decision-making.

## Methods

### Specimens

**Fig. 1:**
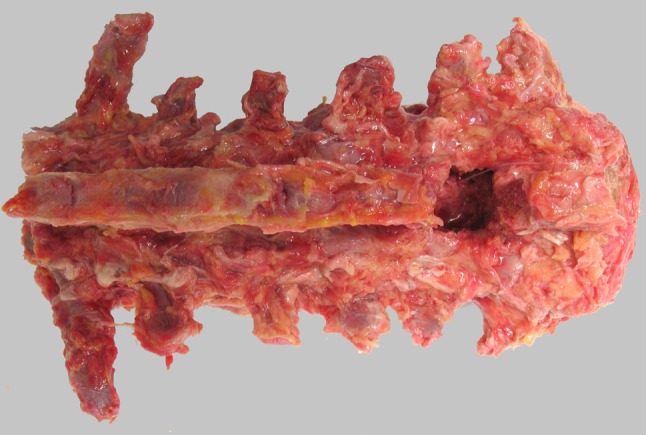
Human thoracolumbar spine (T12–L5) with laminectomy at level L4

Thoracolumbar spines (T12–L5) were harvested from freshly frozen (−20°C) human cadavers (mean age 72.1 years, range 53–89 years). None of the deceased subjects had any history of spinal injury, spinal surgery or spinal metastatic disease. The spines were thawed before assessment and biomechanical testing. Excessive soft tissue and muscle tissue were carefully removed, keeping the anterior and posterior longitudinal ligaments as well as the facet joints intact (Fig. 1).

### Parameters

For assessment of spines, we used clinically relevant and methodologically validated parameters of lumbar spinal degeneration as recommended by the European Spine Society [9]. Grading methods for disc degeneration with an intraclass correlation coefficient or an interobserver κ > 0.60 [5, 13, 17] were included. For facet joint degeneration, grading schemes [9] with an intraclass correlation coefficient or interobserver κ > 0.40 were used in the present study [16, 26].

Magnetic resonance imaging (MRI, Siemens© Symphony 1.5 T: Syngo MR A30, software NUMARIS/4, Berlin, Germany) of lumbar spines was performed to assess intervertebral disc degeneration according to Griffith and Pfirrmann [5, 17] and facet joint degeneration according to Weishaupt [26]. Disc degeneration, (including narrowing and osteophytes, respectively Lanes 1 and 2) [13, 27] and facet joint degeneration [16] of levels L2–L3 and L4–L5 were also assessed based on radiographs (Sedical© Digital Vet. DX-6, Arlington Heights, IL, USA). Furthermore, MRI was used to assess the presence of Modic changes [15] and Schmorl’s nodes [18] and to determine intervertebral disc and pedicle geometry and facet joint angles [2]. Disc geometry included: disc length, width, height, surface area, and volume. Disc surface area, disc volume and pedicle diameter were calculated assuming an elliptic shape (surface = 1/4π × length × width). For pedicle diameter, an average of left and right pedicles was taken for the top (L2 or L4) and bottom (L3 or L5) of each segment. Mean facet joint angle was calculated by averaging left and right angles per segmental level (L2–L3 or L4–L5) while facet joint angle differences or tropism was determined by calculating the difference between left and right facet joint angles. Segmental frontal surface area (FA), defined in cm^2^, bone mineral content (BMC in g) and bone mineral density (BMD in g/cm^2^) of lumbar spinal sections (L2–L3 and L4–L5) were measured with dual X-ray absorptiometry (DXA, Hologic© QDR 4500 Delphi DXA scanner, Waltham, MA, USA) in anteroposterior direction. All assessments were performed using Osirix software (Osirix©, version 3.8.1., Pixmeo SARL, Geneva, Switzerland).

### Specimen preparation and biomechanical testing

L2–L3 and L4–L5 motion segments were isolated from each spine. Subsequently, laminectomy was performed at level L2 of five randomly chosen spines, and at level L4 of the remaining five spines. Laminectomy, analogous to standard clinical practice, was performed by removing the spinous process and part of the lamina, leaving the pars interarticularis intact. During preparation, examination, and biomechanical testing, specimens were kept hydrated using 0.9% saline-soaked gauzes. Thoracolumbar spines with bridging osteophytes, assessed on anteroposterior, lateral and oblique radiographs, were excluded from this study. After sectioning spines in L2–L3 and L4–L5 motion segments, the motion segments were potted in a casting-mould using low melting point (48°C) bismuth alloy (Cerrolow-147; 48.0% bismuth, 25.6% lead, 12% tin, 9.6% cadmium, and 4% indium). The upper and lower vertebral bodies were fixed securely into the alloy by adding screws into the vertebral body. Screw fixation was reinforced with orthopaedic bone cement (Simplex, Stryker©, Kalamazoo, MI, USA). The disc was placed parallel to the flat surface of the bismuth. Discs were placed parallel based on the visual inspection. Because muscle tissue was thoroughly and carefully removed, the intervertebral disc and corresponding endplates were clearly visible. All articulating parts were kept free. The casting mould was placed in a hydraulic materials testing machine (Instron©, model 8872, Norwood, Canada) [1, 23, 24]. The caudal vertebral body was fixed on a plateau that allowed movement in axial and transverse directions only. Transverse movements were allowed, so segments were able to find their physiological motion patterns and to correct for possible differences in embedding. Segments were loaded with a continuous axial compressive force of 1600 N [23, 24], applied using a pneumatic cylinder that had been calibrated using a load cell (Hottinger Baldwin Messtechnik©, Force Transducer Type C2, Darmstadt, Germany). Since compression was applied in a purely axial direction, bending moments were minimized. The level of compression simulated the force during bending, a condition in which high shear loading of the lumbar spinal segments typically occurs [23]. Subsequently, while maintaining the axial load, anterior shear load was applied with a constant rate of 2.0 mm/min on the casting mould containing the cranial vertebral body, until failure of the vertebral motion segment [24]. This test set-up was similar to mechanical testing by Bisschop et al. [1], van Solinge et al. [24] and van Dieën et al. [23]. An anterior shear force was used since it corresponds to the loading direction in vivo [10–12, 22]. The test was stopped after hearing a crack or after a large force reduction was seen. Shear force and displacement were digitized and stored at 100 samples per second (Instron© Fast Track 2, Norwood, Canada).

For each of the 20 motion segments tested, SFF was determined. SFF was defined as the point at which maximum load was recorded in the load–displacement curves for each specimen. These data were presented previously [1]. Shear yield force (SYF) was defined as the point at which shear load caused a decrease in stiffness, i.e. a decrease in the slope of the load–displacement curve. Average SS was calculated from the load–displacement curve, between 25 and 50% of the SFF. SS was estimated by means of a least squares fit of a straight line through the data with the slope of the regression line representing stiffness. The deformation in this region was linear, with an *r* ^2^ > 0.943 (Table 1) between load and displacement for all motion segments. All analyses were performed using computer programs written in Matlab (Mathworks ©, Natick, MA, USA).

**Table 1:** Overview of specimens and biomechanical outcomes per tested segment

	Segment	Laminectomy	Shear stiffness	Shear yield force	Shear force to failure^a^
		(0/1)	(SS)	(SYF)	(SFF)
			(N/mm)	(N)	(N)
Specimen 01 male, 79	L2–L3	0	327 (0.998)	1,052	2,317
	L4–L5	1	159 (0.995)	1,045	1,258
Specimen 02 male, 53	L2–L3	0	213 (0.995)	1,527	3,284
	L4–L5	1	247 (0.993)	1,137	1,886
Specimen 03 male, 72	L2–L3	0	232 (0.943)	967	1,678
	L4–L5	1	307 (0.999)	815	1,775
Specimen 04 female, 82	L2–L3	0	214 (0.988)	888	909
	L4–L5	1	342 (0.999)	390	561
Specimen 05 male, 78	L2–L3	0	252 (0.998)	1,100	1,292
	L4–L5	1	211 (0.997)	867	1,221
Specimen 06 male, 79	L2–L3	1	162 (0.998)	431	994
	L4–L5	0	378 (0.994)	1,136	2,408
Specimen 07 male, 62	L2–L3	1	200 (0.995)	420	940
	L4–L5	0	273 (0.991)	1,212	2,724
Specimen 08 female, 64	L2–L3	1	217 (0.999)	304	660
	L4–L5	0	236 (0.996)	1,083	1,553
Specimen 09 female, 63	L2–L3	1	64 (0.967)	278	641
	L4–L5	0	308 (0.995)	1,135	1,313
Specimen 10 female, 89	L2–L3	1	178 (0.995)	709	721
	L4–L5	0	309 (1.000)	774	1,628

For shear stiffness, *r* ^2^ values are added in brackets

*0* untreated, *1* laminectomy

^a^Presented previously

### Statistical methods

Statistical analysis was performed based on two separate groups. The first group contained untreated segments (5× L2–L3 and 5× L4–L5) while the second group consisted of segments with laminectomy (5× L2–L3 and 5× L4–L5).

Independent variables were classified as: general variables, intervertebral disc geometry (MRI), pedicle geometry (MRI), facet joint orientation (MRI), bone characteristics (DXA), intervertebral disc degeneration classifications (MRI), intervertebral disc and facet joint degeneration (Radiographs), facet joint degeneration (MRI) and other (MRI). These classes of variables are specified in Table 2.

**Table 2:** Overview of correlations (*p* values, two tailed <0.05: in bold) between independent and dependent variables in untreated and treated segments

			Untreated			Laminectomy		
			SS	SYF	SFF	SS	SYF	SFF
*General variables*								
Segment	DV	–	−1.69 (0.129)	0.29 (0.781)	−0.06 (0.953)	−2.10 (0.069)	−**2.82** (**0.023**)	−2.23 (0.057)
Sex	CV	–	0.33 (0.751)	1.63 (0.141)	**2.41** (**0.043**)	0.27 (0.796)	2.11 (0.068)	**3.43** (**0.009**)
Age	CV	–	0.39 (0.269)	−**0.84** (**0.002**)	−0.51 (0.132)	0.09 (0.800)	0.00 (0.992)	−0.35 (0.328)
*Intervertebral disc geometry*								
Disc length	CV	MRI	−0.11 (0.773)	0.05 (0.898)	0.38 (0.277)	0.40 (0.255)	**0.66** (**0.039**)	**0.73** (**0.016**)
Disc width	CV	MRI	−0.02 (0.947)	0.06 (0.877)	0.38 (0.283)	0.24 (0.501)	0.13 (0.715)	−0.02 (0.964)
Disc height	CV	MRI	0.06 (0.875)	0.39 (0.272)	0.23 (0.518)	−0.51 (0.131)	0.34 (0.337)	0.21 (0.566)
Disc surface	CV	MRI	−0.08 (0.822)	0.05 (0.891)	0.37 (0.292)	0.56 (0.090)	**0.66** (**0.037**)	0.63 (0.052)
Disc volume	CV	MRI	0.05 (0.881)	0.41 (0.237)	0.43 (0.220)	−0.17 (0.647)	**0.68** (**0.032**)	0.51 (0.130)
*Pedicle geometry*								
Sections top (L2 or L4)	CV	MRI	−0.01 (0.977)	−0.50 (0.143)	−0.12 (0.741)	0.53 (0.116)	0.52 (0.121)	0.26 (0.467)
Sections bottom (L3 or L5)	CV	MRI	−0.15 (0.683)	−**0.83** (**0.003**)	−0.52 (0.122)	0.43 (0.219)	0.46 (0.183)	0.07 (0.850)
*Facet joint orientation*								
Mean facet joint angle	CV	MRI	−0.37 (0.291)	0.15 (0.676)	0.36 (0.312)	−0.40 (0.256)	0.34 (0.345)	0.03 (0.933)
Facet joint tropism	CV	MRI	**0.71** (**0.022**)	−0.19 (0.594)	0.35 (0.329)	0.44 (0.207)	−0.11 (0.772)	−0.18 (0.617)
*Bone characteristics*								
Frontal area	CV	DXA	0.61 (0.063)	0.39 (0.260)	**0.70** (**0.024**)	0.44 (0.208)	**0.75** (**0.012**)	0.61 (0.059)
Bone mineral content	CV	DXA	0.09 (0.799)	0.61 (0.064)	**0.85** (**0.002**)	0.13 (0.721)	**0.65** (**0.041**)	**0.81** (**0.005**)
Bone mineral density	CV	DXA	−0.05 (0.900)	0.54 (0.109)	**0.75** (**0.013**)	−0.06 (0.875)	0.33 (0.346)	0.63 (0.052)
*Intervertebral disc degeneration*								
Pfirrmann	OV	MRI	−0.04 (0.908)	−0.56 (0.092)	−0.27 (0.451)	**0.64** (**0.045**)	0.19 (0.601)	0.09 (0.798)
Griffith	OV	MRI	−0.13 (0.731)	−0.37 (0.288)	−0.16 (0.668)	**0.70** (**0.026**)	0.16 (0.659)	0.14 (0.699)
*Intervertebral disc and facet joint degeneration*								
Lane-1 (Narrowing)	OV	Radiographs	−0.18 (0.618)	−0.43 (0.211)	−0.36 (0.304)	0.56 (0.094)	0.10 (0.780)	0.07 (0.848)
Lane-2 (Osteophytes)	OV	Radiographs	−0.17 (0.643)	−0.24 (0.509)	−0.05 (0.896)	**0.73** (**0.017**)	0.14 (0.698)	0.12 (0.733)
Wilke	OV	Radiographs	−0.18 (0.618)	−0.43 (0.211)	−0.36 (0.304)	0.56 (0.094)	0.10 (0.780)	0.07 (0.848)
Pathria	OV	Radiographs	0.29 (0.422)	−0.49 (0.153)	−0.55 (0.102)	**0.64** (**0.044**)	−0.15 (0.681)	−0.08 (0.833)
*Facet joint degeneration*								
Weishaupt	OV	MRI	0.37 (0.299)	−0.43 (0.220)	−0.57 (0.084)	0.51 (0.129)	0.09 (0.812)	−0.07 (0.843)
*Other*								
Schmorl’s nodes	DV	MRI	1.20 (0.263)	−0.44 (0.671)	0.56 (0.589)	−0.22 (0.831)	0.24 (0.820)	0.16 (0.880)
Modic changes	DV	MRI	−0.74 (0.480)	−0.17 (0.869)	−1.00 (0.345)	0.60 (0.565)	0.68 (0.519)	0.15 (0.881)

For DVs, *t* values are presented while correlations based on CVs and OVs are described by Pearson’s coefficient of correlation

*SS* shear stiffness, *SYF* shear yield force, *SFF* shear force to failure, *DV* dichotomized variable, *CV* continuous variable, *OV* ordinal variable

First, relations between independent and dependent variables (SS, SYF and SFF) were tested for each individual variable. For dichotomized independent variables (segment, sex, Modic changes [15] and Schmorl’s nodes [18], independent-sample *t* tests were used while Pearson’s coefficient of correlation was determined for continuous and ordinal values. Note that it was thus assumed that ordinal variables (Pfirrmann [17], Griffith [5], Lane 1 [13], Lane 2 [13], Wilke [27], Pathria [16] and Weishaupt [26]) represent a linear degree of severity.

When independent variables were associated with a dependent variable, here defined as independent-sample *t* test: *p* < 0.05 or as a bivariate correlation with a significance level of: *p* < 0.05, they were used for the combined statistical models.

Before final analysis was performed, all independent variables were checked for correlations with each other. In case a correlation >0.7 with a *p* < 0.05 was found, the independent variable with the strongest effect on the specific dependent variable was included in the model. Finally, backward linear regression techniques were used to create final statistical models per dependent variable per treatment group.

## Results

**Fig. 2:**
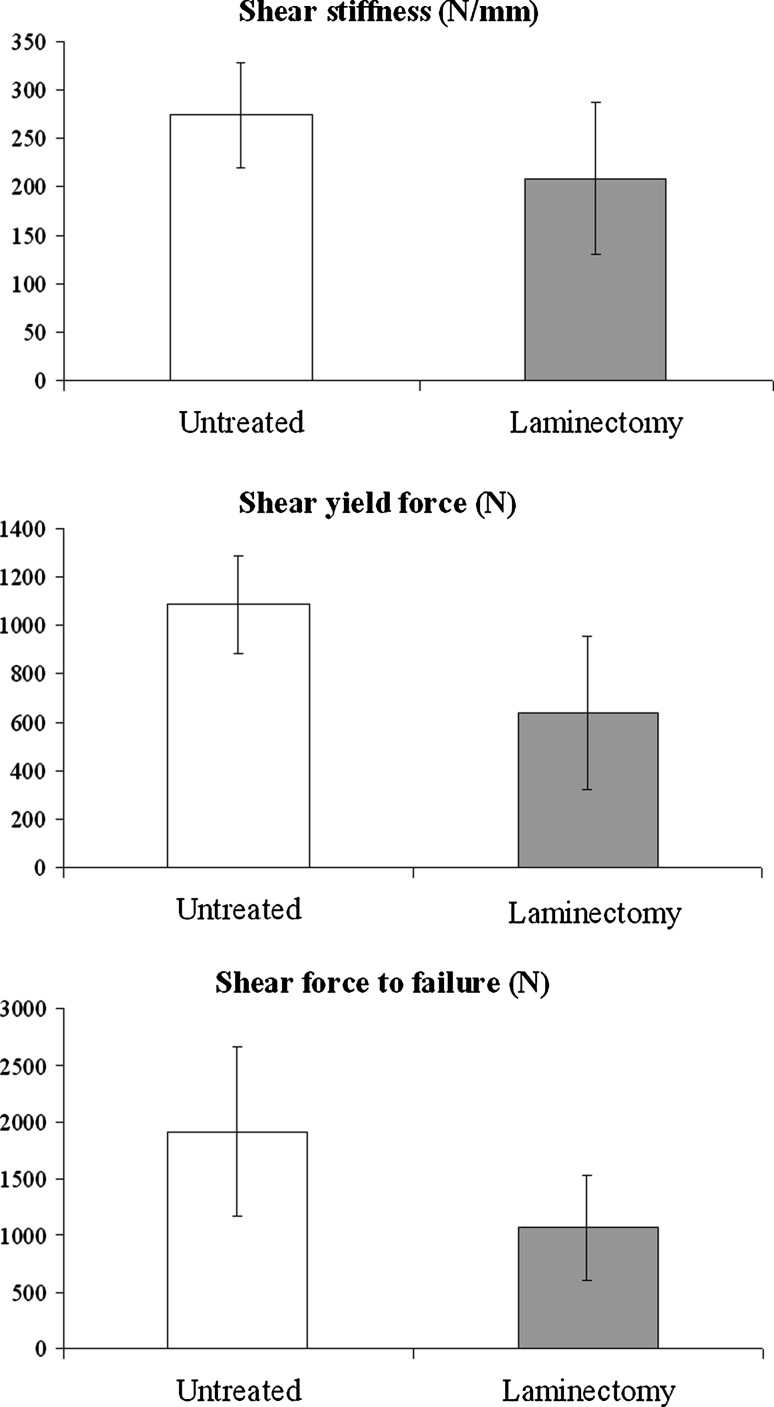
Effects of lumbar laminectomy on shear biomechanics, showing a substantial decrease of shear stiffness (23.7%), shear yield force (41.1%) and shear force to failure (44.3%) following laminectomy

All specimen characteristics and biomechanical properties for segments with and without laminectomy are presented in Table 1. As shown in Fig. 2, lumbar laminectomy had a substantial declining effect on SS (23.7%), SYF (41.1%) and SFF (44.3%).

Table 2 gives an overview of correlations between independent and dependent variables of segments with and without laminectomy. Some of the general variables, as presented in Table 1 (sex and age for untreated segments, and segment level and sex for treated segments), were related to strength parameters (SYF and/or SFF) in both groups. In the untreated segments, SFF was found to be lower for female specimens (2.284 N for male versus 1.351 N for female). For the treated segments, SFF was also found to be lower for female specimens (1.346 N for male versus 646 N for female). SYF in treated segments proved to be level dependent (L2–L3: 428 N versus L4–L5: 851 N).

For segments treated with laminectomy, three out of five intervertebral disc geometry variables (i.e., length, surface and volume) were significantly related to biomechanical shear properties (SYF; all three, for SFF; length only). In contrast, biomechanics of untreated segments were unrelated to intervertebral disc geometry. The opposite was true for pedicle geometry and facet joint orientation. Pedicle sections and facet joint angle difference correlated significantly to respectively, SYF and SS in untreated segments but did not correlate with biomechanical outcomes in treated segments.

For both groups, bone characteristics measured with DXA, were strongly related to shear strength parameters (SYF and SFF), but not to stiffness (SS). Like intervertebral disc geometry, intervertebral disc degeneration was predictive for biomechanics (SS) of spinal segments with laminectomy. This was consistent over imaging methods and classification schemes (MRI; Pfirrmann [17] and Griffith [5] or radiographs; Wilke [27]), although not significant for radiographs (correlation: 0.558, *p* value: 0.094). However, in contrast to intervertebral disc geometry, these intervertebral disc degeneration scores were not related to strength (SYF and SFF). Finally, Modic changes [15] and Schmorl’s nodes [18] were not related to shear biomechanics of spinal segments with or without laminectomy.

Results of the backward linear regression, using determinants of spine biomechanics, which were identified (based on a *p* < 0.05) in Table 2, are presented in Table 3. All models, describing strength parameters (SYF and SFF) consisted of two independent variables.

**Table 3:** Overview of backward linear regression models per dependent variable in untreated and in treated segments based on significant correlation coefficients found in Table 2

Untreated				
* Shear stiffness*	*Variables:*	*Constant*	*Facet joint tropism*	
N/mm	Factor:	204	15	
*r* ^2^ value: 0.50	Significance:	>0.001	0.022	
* Shear yield force*	*Variables:*	*Constant*	*Age*	*Pedicle section bottom*
N	Factor:	2,102	−9	−418
*r* ^2^ value: 0.83	Significance:	>0.001	0.050	0.064
*Shear force to failure*	*Variables:*	*Constant*	*Frontal area*	*Bone mineral content*
N	Factor:	−2,317	82	55
*r* ^2^ value: 0.83	Significance:	0.122	0.093	0.008
Laminectomy				
* Shear stiffness*	*Variables:*	*Constant*	*Lane-2 (osteophytes)*	
N/mm	Factor:	166	48	
*r* ^2^ value: 0.53	Significance:	>0.001	0.017	
*Shear yield force*	*Variables:*	*Constant*	*Segment*	*Disc volume*
N	Factor:	−47	363	25
*r* ^2^ value: 0.81	Significance:	0.773	0.008	0.011
* Shear force to failure*	*Variables:*	*Constant*	*Sex*	*Disc length*
N	Factor:	286	−494	234
*r* ^2^ value: 0.77	Significance:	0.570	0.035	0.058

Each row in the table represents a regression equation. Models were based on the highest statistical power, using backward linear regression techniques

SYF and SFF could accurately be predicted by the final statistical model for untreated segments (*r* ^2^ value respectively: 0.83; 0.83). Age and pedicle geometry remained in the model for SYF, while for SFF, the final model consisted of DXA parameters (frontal area and BMC) only. For segments treated with laminectomy, SYF and SFF could be predicted from independent variables with *r* ^2^ values of 0.81 (intervertebral disc volume and segment) and 0.77 (sex and intervertebral disc length), respectively. SS was less accurately predicted with only a single variable remaining in the model for both untreated segments (facet joint angle difference; *r* ^2^ = 0.50) and segments with laminectomy (degeneration score Lane-2 (osteophytes); *r* ^2^ = 0.53).

## Discussion

The aim of this study was to identify parameters, that predict spinal shear properties before and after laminectomy, in order to determine which of these parameters may prognosticate spinal instability following lumbar laminectomy.

For characterization of the spinal motion segments, we used commonly applied grading systems to assess disc degeneration [5, 13, 17, 27], facet joint degeneration [9, 16, 26], Modic changes [15] and Schmorl’s nodes [18] based on MRI and radiographs. Furthermore, we measured intervertebral disc and pedicle geometry, facet joint angles [2] and bone characteristics (BMC: bone mineral content; BMD and total segmental surface area on DXA defined as frontal area: FA). These parameters all potentially affect strength and SS of the lumbar spinal motion segment before and/or after treatment with laminectomy and can be determined in clinical practice.

We showed that multiple variables are related to spinal shear properties in intact lumbar segments and lumbar segments treated with laminectomy. Statistical models with these parameters as independent variables predicted shear biomechanics, with moderate to very good accuracy with *r* ^2^ values varying from 0.50 to 0.83 (without laminectomy) and from 0.53 to 0.81 (with laminectomy). Particularly, strength parameters (SYF and SFF) in both untreated and treated segments could be predicted with good to very good accuracy. Prediction of SS was only moderately accurate.

The tests on individual variables (Table 2) showed that, for untreated segments, pedicle geometry was related to SYF and facet joint orientation to SS. In contrast, for segments with laminectomy, intervertebral disc characteristics appeared to determine shear properties. Intervertebral disc characteristics correlated to strength and disc degeneration correlated to stiffness. For both segments with and without laminectomy, DXA assessment was found to be important, although mainly for strength parameters.

SYF might be the most critical shear property, because it marks the beginning of the irreversible deformation of a spinal motion segment, signalling the appearance of the first soft tissue and or trabecular bone lesions [21]. We expect that when shear loading crosses the yield point, sub-clinical damage will occur. Such damage may, at a later stage, lead to symptomatic spondylolisthesis. Unlike SYF, SFF marks, as the description suggests, complete and irreversible failure of spinal motion segments. SFF describes an acute clinically relevant situation. Therefore, SYF and SFF represent different clinical value. In untreated segments SYF depended mainly on pedicle geometry, while SFF strongly correlated with DXA parameters (Table 2). For treated segments, both SYF and SFF were correlated with intervertebral disc geometry and DXA parameters (Table 2) and both parameters could be predicted quite accurately (Table 3).

In this study, SS was only moderately predictable (*r* ^2^ values: 0.50 and 0.53). We assumed that the intervertebral disc has a large contribution to this biomechanical parameter. This assumption was corroborated by the results (Table 2). Degenerative parameters proved to be strongly correlated with SS in treated segments. In other words, laminectomy leads to a shift in load bearing, from the pars interarticularis to the intervertebral disc. Unfortunately, we could only study the morphology and degeneration of the intervertebral disc on MRI and radiographic imaging. A more specific (histological) analysis of the state of the intervertebral disc may strengthen correlations [19], but may not be clinically applicable.

For stiffness, *r* ^2^ values of only 0.50 and 0.53 were found. As stated earlier, stiffness was mainly determined by degenerative parameters, such as disc degeneration. The fact that these parameters are based on visual assessment and have an ordinal character possibly explains their lower predictive value, compared to directly measured continuous variables such as BMD and BMC.

In our protocol, both BMD and BMC were studied. BMD is often used as a clinical parameter. However, BMC, can also be used to express the bone mineral content since it integrates information on bone density and vertebral dimensions. BMC is defined as BMD (g/cm^2^) multiplied by the total segmental surface area (FA) of the spinal segment (cm^2^) and is expressed in grams. We therefore decided to include both parameters as a factor that prognosticates instability following lumbar laminectomy.

We found a substantial difference between male and female specimen considering SFF, in both treated and untreated segments. However, considering the limited number of tested specimens, we cannot draw any conclusions from these findings.

In vivo, muscle forces are very important [20]. Muscle forces are the main generators of compression and shear forces. We simulated the effect of muscle forces on the spine using static 1600 N compressive force and an increasing shear force imposed by the material testing machine. The chosen preload of 1600 N was selected to allow for comparison with previous work [23, 24] and was a compromise between applying compression forces that are sufficiently large to simulate spinal loads that occur in vivo when large shear forces are present [10–12, 22], but low enough to avoid damage due to compression forces alone [3].

One limitation of this study is that small alignment errors may have been present. Our results, however, are not likely to be very sensitive to small errors in specimen alignment. Previously, it was shown that SS and SFF were not different between specimens in neutral position and specimen in 10° of flexion [23]. Therefore, we do not expect significant changes in biomechanical outcomes when malaligning segments.

Another limitation of this study was that we did not investigate the nature of failure. Van Solinge et al. [24] investigated types of failure, occurring with shear loading. These failure mechanisms were similar to those found in clinical practice. Since our test setup was similar, we expect our segments to fail in similar fashion.

From a clinical point of view, laminectomy at a spinal segment that exhibits small intervertebral disc geometry, disc and facet joint degeneration and poor bone mineral density may need additional instrumental spinal stabilization to reduce the risk of post-operative instability. However, also pull out strength of spinal implants, proved to be dependent on bone mineral quality as measured by dual X-ray absorptiometry (DXA) [25] and this dependency needs to be taken into account when deciding on instrumentation.

Considering further research, we recommend to assess the parameters found to be predictive in a prospective or retrospective in vivo design. In addition to shear failure, further studies should also focus on other failure mechanisms of the human lumbar spine, including axial rotation [4].

Finally, while *r* ^2^ values, as we presented, may be too low to provide the sole basis for decisions upon surgical stabilization after laminectomy. Strength parameters (SYF and SFF) correlations were predicted with reasonable accuracy (*r* ^2^ values between 0.77 and 0.83). As currently surgeons decide based upon personal experience, a more informed choice might benefit this decision.

In conclusion, predictive models with moderate to good accuracy were found for SYF and SFF of human lumbar spinal segments with and without laminectomy. Significant loss of SS and strength are predicted by BMC, BMD, intervertebral disc geometry and degenerative parameters. Therefore, knowledge of a patient’s BMC, BMD, intervertebral disc geometry and the possible presence of osteophytes, might provide valuable information as predictors of the development of post-operative instability following lumbar laminectomy. Pedicle sections and facet geometry were not predictive for the possible development of post-operative instability following lumbar laminectomy.

## References

[1] Bisschop A, Mullender MG, Kingma I, Jiya TU, van der Veen AJ, Roos JC et al. (2011) The impact of bone mineral density and disc degeneration on shear strength and stiffness of the lumbar spine following laminectomy. Eur Spine J (Epub ahead of print) 10.1007/s00586-011-1968-2 PMC3296849 21863461

[2] Boden SD, Riew KD, Yamaguchi K, Branch TP, Schellinger D, Wiesel SW (1996). Orientation of the lumbar facet joints: association with degenerative disc disease. J Bone Joint Surg Am.

[3] Brinckmann P, Biggemann M, Hilweg D (1989). Prediction of the compressive strength of human lumbar vertebrae. Spine (Phila Pa 1976).

[4] Farfan HF (1984). The torsional injury of the lumbar spine. Spine (Phila Pa 1976).

[5] Griffith JF, Wang YX, Antonio GE, Choi KC, Yu A, Ahuja AT (2007). Modified Pfirrmann grading system for lumbar intervertebral disc degeneration. Spine (Phila Pa 1976).

[6] Hansraj KK, Cammisa FP, O’Leary PF, Crockett HC, Fras CI, Cohen MS (2001). Decompressive surgery for typical lumbar spinal stenosis. Clin Orthop Relat Res.

[7] Herkowitz HN, Kurz LT (1991). Degenerative lumbar spondylolisthesis with spinal stenosis. A prospective study comparing decompression with decompression and intertransverse process arthrodesis. J Bone Joint Surg Am.

[8] Jansson KA, Nemeth G, Granath F, Blomqvist P (2005). Spinal stenosis re-operation rate in Sweden is 11% at 10 years—a national analysis of 9,664 operations. Eur Spine J.

[9] Kettler A, Wilke HJ (2006). Review of existing grading systems for cervical or lumbar disc and facet joint degeneration. Eur Spine J.

[10] Kingma I, Bosch T, Bruins L, van Dieen JH (2004). Foot positioning instruction, initial vertical load position and lifting technique: effects on low back loading. Ergonomics.

[11] Kingma I, Faber GS, Bakker AJ, van Dieen JH (2006). Can low back loading during lifting be reduced by placing one leg beside the object to be lifted?. Phys Ther.

[12] Kingma I, Staudenmann D, van Dieen JH (2007). Trunk muscle activation and associated lumbar spine joint shear forces under different levels of external forward force applied to the trunk. J Electromyogr Kinesiol.

[13] Lane NE, Nevitt MC, Genant HK, Hochberg MC (1993). Reliability of new indices of radiographic osteoarthritis of the hand and hip and lumbar disc degeneration. J Rheumatol.

[14] Leone A, Guglielmi G, Cassar-Pullicino VN, Bonomo L (2007). Lumbar intervertebral instability: a review. Radiology.

[15] Modic MT, Steinberg PM, Ross JS, Masaryk TJ, Carter JR (1988). Degenerative disk disease: assessment of changes in vertebral body marrow with MR imaging. Radiology.

[16] Pathria M, Sartoris DJ, Resnick D (1987). Osteoarthritis of the facet joints: accuracy of oblique radiographic assessment. Radiology.

[17] Pfirrmann CW, Metzdorf A, Zanetti M, Hodler J, Boos N (2001). Magnetic resonance classification of lumbar intervertebral disc degeneration. Spine (Phila Pa 1976).

[18] Pfirrmann CW, Resnick D (2001). Schmorl nodes of the thoracic and lumbar spine: radiographic-pathologic study of prevalence, characterization, and correlation with degenerative changes of 1,650 spinal levels in 100 cadavers. Radiology.

[19] Quint U, Wilke HJ (2008). Grading of degenerative disk disease and functional impairment: imaging versus patho-anatomical findings. Eur Spine J.

[20] Quint U, Wilke HJ, Loer F, Claes L (1998). Laminectomy and functional impairment of the lumbar spine: the importance of muscle forces in flexible and rigid instrumented stabilization—a biomechanical study in vitro. Eur Spine J.

[21] Renau A, Farrerons J, Yoldi B, Gil J, Proubasta I, Llauger J (2004). Yield point in prediction of compressive behavior of lumbar vertebral body by dual-energy X-ray absorptiometry. J Clin Densitom.

[22] van Dieen JH, Kingma I (2005). Effects of antagonistic co-contraction on differences between electromyography based and optimization based estimates of spinal forces. Ergonomics.

[23] van Dieen JH, van der Veen AJ, van Royen BJ, Kingma I (2006). Fatigue failure in shear loading of porcine lumbar spine segments. Spine (Phila Pa 1976).

[24] van Solinge GB, van der Veen AJ, van Dieen JH, Kingma I, van Royen BJ (2010). Anterior shear strength of the porcine lumbar spine after laminectomy and partial facetectomy. Eur Spine J.

[25] van Laar W, Meester RJ, Smit TH, van Royen BJ (2007). A biomechanical analysis of the self-retaining pedicle hook device in posterior spinal fixation. Eur Spine J.

[26] Weishaupt D, Zanetti M, Boos N, Hodler J (1999). MR imaging and CT in osteoarthritis of the lumbar facet joints. Skeletal Radiol.

[27] Wilke HJ, Rohlmann F, Neidlinger-Wilke C, Werner K, Claes L, Kettler A (2006). Validity and interobserver agreement of a new radiographic grading system for intervertebral disc degeneration: part I. Lumbar spine. Eur Spine J.

